# Development of Postpartum Depression in Pregnant Women with Preeclampsia: A Retrospective Study

**DOI:** 10.1155/2019/9601476

**Published:** 2019-02-27

**Authors:** Li Chen, Xiaodan Wang, Qian Ding, Nan Shan, Hongbo Qi

**Affiliations:** ^1^The Department of Obstetrics, The First Affiliated Hospital of Chongqing Medical University, Chongqing 400016, China; ^2^State Key Laboratory of Maternal and Fetal Medicine of Chongqing Municipality, Chongqing Medical University, Chongqing 400016, China; ^3^International Collaborative Laboratory of Reproduction and Development of Chinese Ministry of Education, Chongqing Medical University, Chongqing 400016, China; ^4^The Department of Dermatology and Venereology, The First Affiliated Hospital of Chongqing Medical University, Chongqing 400016, China

## Abstract

**Background:**

Postpartum depression (PPD) and preeclampsia (PE) are both common diseases in obstetrics that affect maternal health and infant development. However, the relationship between the two diseases still requires clarification.

**Objective:**

The purpose of this study was to (1) determine the incidence rate of PPD in patients with PE and (2) identify the association between the prevalence of PPD and the severity of PE.

**Methods:**

We conducted a retrospective analysis of women with and without PE who delivered between January 1, 2017, and August 30, 2018, in the First Affiliated Hospital of Chongqing Medical University. We used a questionnaire survey methodology that included the Edinburgh Postnatal Depression Scale (EPDS) to test the influence of PE on the development of new-onset PPD in the 6 weeks after delivery. We determined PPD based on a score ≥10 on the EPDS. Bivariate analysis was used to compare data between the two groups.

**Results:**

A total of 180 women participated in this study. Thirty-five people screened positive for PPD, while the remaining 145 screened negative. The prevalence of PPD was 26.67% (24/90) in patients with PE, which was two times the prevalence in normal women (12.22%). Multiple logistic regression showed that women who had PE had nearly 3-fold increased odds of PPD compared to normal women and the risk of PPD increased with the aggravation of PE. Patients with severe PE had a more than 4-fold increased risk of screening positive for PPD.

**Conclusion:**

PE was independently associated with PPD. Furthermore, the risk of PPD seemed to increase with the aggravation of PE. Thus, additional prevention efforts and support methods should be provided for women with PE to reduce the incidence of PPD.

## 1. Introduction

Puerperae experience pathophysiological changes and role shifts after childbirth and become more prone to depression than during other periods of life [1]. The incidence of postpartum depression (PPD) varies greatly among patients in different regions, of different races and with different economic backgrounds, with a prevalence range of approximately 6.5-19% [2, 3]. The morbidity is higher when women have cesarean section deliveries, are underweight, are obese, have pelvic floor symptoms, and experience other special situations [4–6]. PPD has a negative influence on women's health, family harmony, and infant development and has become a public health problem worldwide.

Preeclampsia (PE) is a common obstetric complication with an incidence of approximately 3-5% in all pregnant women [7]. At the same time, it is also a severe obstetric disease associated with serious maternal-fetal morbidity and mortality [8]. The pathogenesis of PE is still unclear and may be related to hypoperfusion and ischemia of the placenta. The ischemic placenta will cause oxidative stress and release inflammatory factors, thus leading to systemic endothelial dysfunction. The maternal consequences include disseminated intravascular coagulation, hypertensive encephalopathy, heart failure, thrombocytopenia, renal failure, and HELLP syndrome. Fetal consequences of PE include preterm labor, fetal distress, and fetal growth restriction [9].

Studies have shown that hypertension is a risk factor for depression; in hypertensive patients, the prevalence of depression is as high as 26.8% [10, 11]. Although chronic hypertension is slightly different from PE in patients in terms of pathogenesis and some clinical symptoms, the manifestations of both diseases are mainly elevated blood pressure and damage to vital organs, such as the heart, brain, and kidneys. However, it is unknown whether there is a link between PE and PPD, given that they are two common conditions in obstetrics. One study that investigated obstetric risk factors for depression mentioned that PE increased the incidence of PPD [12]. However, few articles have systematically researched the relationship between PE and PPD.

In our study, we collected data from eligible pregnant women who delivered between January 1, 2017, and August 30, 2018, in the First Affiliated Hospital of Chongqing Medical University. The purpose of this study was to (1) determine the incidence rate of PPD in patients with PE and (2) identify whether the prevalence of PPD will increase with the aggravation of PE.

## 2. Materials and Methods

We obtained written informed consent from all subjects included in the research. We performed a retrospective study of women who delivered between January 1, 2017, and August 30, 2018, in the First Affiliated Hospital of Chongqing Medical University. All women were required to follow up in a specialty postpartum clinic approximately 6 weeks after delivery.

Women who met the inclusion criteria were included in our research. The inclusion criteria were as follows: (1) either being diagnosed with PE based on the clinical guidelines published by ACOG in 2013 [8] or not being diagnosed with PE and being part of the control group; (2) age between 20 to 40 years old; (3) gestational age≥28 weeks; and (4) vaginal delivery or cesarean section and neonatal survival. The exclusion criteria were as follows: (1) having obstetric complications other than PE; (2) having a previous history of PPD or anxiety, or a family history of depression, or other mental disorders; and (3) having a stillborn fetus or lethal induction of labor. Patients who met the inclusion and exclusion criteria were asked to fill in a questionnaire survey when they came to the specialty postpartum clinic approximately 6 weeks after delivery. The questionnaire survey collected information on the last 7 days and included graded questions (e.g., “What has been the range of your blood pressure fluctuations in the last 7 days?”) and “yes/no” questions (e.g., “Have you taken any medicine to control your blood pressure in the last 7 days?”). Data on demographic characteristics, mode of delivery, and BMI before delivery were collected from the electronic medical records system in our institution.

The Edinburgh Postnatal Depression Scale (EPDS) is a self-evaluation scale that consists of 10 items. Each item is divided into 4 grades and is scored from 0 to 3. The total score ranges from 0 to 30, with higher scores signifying more serious PPD [13]. Based on the original scale, Chinese experts modified the language expression and choice format of each item to make the expression more consistent with the linguistic habits of the mainland Chinese population [14]. The content validity radio (CVR) of the Chinese edition of the EPDS is 0.9333, and the internal consistency reliability is supported by Cronbach's *α* coefficient of 0.76 [14]. The coincidence rate of the EPDS for translation and back-translation is 100% [14]. The EPDS is the most commonly used postnatal depression scale, used in 47.1% of healthcare facilities in China [15]. The proposed threshold for PPD in the Chinese edition is 9.5 points [14]. Therefore, a score on the EPDS ≥10 was determined to be positive for PPD screening in our study.

All women included in our study were divided into PPD and NO-PPD groups according to the results of EPDS screening. The clinical data analysis was performed using SPSS 20.0 (IBM Corporation, Amon City, NY). If continuous variables conformed to a normal distribution, they were presented as the mean ± standard deviation, and an independent sample t-test was used to determine the differences. Otherwise, continuous variables were described by the median ± quartile, and the Mann-Whitney U test was used to determine differences if the variables did not dissatisfy a normal distribution. Categorical data were expressed as frequencies and percentages, and chi-square tests were used to evaluate differences. P <0.05 was considered to be statistically significant. Finally, multiple logistic regression was used to estimate crude odds ratios and adjusted odds ratios.

## 3. Results

A total of 90 patients with PE met the inclusion and exclusion criteria. We randomly selected 90 normal pregnant women who met the inclusion criteria in the same period. Finally, 180 women participated in our study and completed the questionnaire survey. They were divided into PPD and NO-PPD groups according to whether they screened positive for EPDS; their data are summarized in Table 1. As shown in Table 1, a total of 35 people screened positive for PPD, while the remaining 145 screened negative. The differences in age, gestational weeks, gravidity, parity, prior number of cesarean sections, BMI before delivery, mode of delivery, premature birth, and neonatal weight were not significant between the two groups. However, in the PPD group, 24 women had PE with a much higher incidence rate than in the NO-PPD group, in which 66 women suffered from PE (24/35 vs. 66/145, P=0.014). Thus, PE may be a risk factor for PPD.

To further clarify whether the mode of delivery, gestational weeks, or demographic characteristics were risk factors for PPD, we analyzed these data in both normal women and patients with PE. Among the normal women, 11 suffered from PPD, with a prevalence of 12.22% (11/90). There was no significant difference between the two groups in demographic characteristics, mode of delivery, and premature birth rate. The collected data and bivariate analysis in women with and without PPD are shown in Table 2.

On the other hand, 26.67% (24/90) of patients with PE screened positive for PPD, which was two times more than the number of normal puerperae screening positive for PPD. Among the 90 patients, 52 patients had severe PE. The incidence of PPD was 28.85% (15/52) in patients with severe PE. Five patients had slightly higher blood pressure in the previous 7 days, ranging from 140/90 to 160/110 mmHg. Two of these patients screened positive for PPD, and 3 screened negative for PPD. There were 6 patients with PPD and 18 patients without PPD taking drugs (Adalat GITS) for blood pressure control in the previous 7 days. Only one patient with PE occasionally had mild headaches.  Table 3 shows the basic data we collected for patients with PE. Similarly, there was no obvious difference in these data between the PPD group and NO-PPD group.

The average score of the EPDS (4.42 vs. 7.27, *P* < 0.05) was obviously higher in patients with PE compared with normal women. When patients had severe PE, the average score was 8.65 with a significant difference to normal women (Table 4). Finally, we performed multiple logistic regression to estimate the adjusted odds ratio, with PPD as the dependent variable and the presence of PE, gravidity, parity, previous cesarean section, gestational weeks, neonatal weight, BMI before delivery, and mode of delivery as the independent variables (Table 5). The result showed that women who had PE had nearly 3-fold increased odds of PPD compared to normal women. When “severe PE” was used in the model, it had a more than 4-fold increased risk for screening positive for PPD. This finding indicates that the risk of PPD will increase with the aggravation of PE.

## 4. Discussion

To our knowledge, this retrospective study is the first to analyze the relationship between PE and PPD. In our study, the incidence of PPD was 19.44% (35/180), slightly higher than that reported in other articles [2, 3]. Among them, 26.67% of patients with PE screened positive for PPD, which was two times more than the number of normal women screening positive for PPD. With the exception of the postpartum state, people who suffer from hypertension have a higher risk of depression. A meta-analysis that included 41 articles with a total population of 30,796 suggested that the summarized incidence of depression among hypertensive patients is 26.8%, which is similar to our finding [11].

Several mechanisms, such as clinical symptoms, inflammation, and genetic changes, have been used as hypotheses for the relationship between PE and PPD. In our study, although almost all women with PE had no obvious clinical symptoms when they completed the questionnaire survey, most of them suffered from headaches, cutaneous edema, and abnormal liver function several weeks before and after delivery. The clinical symptoms were more serious particularly in patients with severe PE. Of the 15 patients with severe PE and PPD, 10 had severe headaches, 7 had ascites before delivery, and 5 had severe liver dysfunction with levels of serum transaminase more than 5 times higher than their normal levels. These symptoms persisted until approximately 2 weeks after delivery; they may have caused women persistent negative emotional experiences and thus become a trigger for PPD. Conversely, in patients who screened negative for PPD, the severity and duration of clinical symptoms associated with PE were less severe than in the PPD group. Therefore, clinical symptoms may be a hypothesis for the increasing incidence of PPD.

It is well known that the “two-stage theory” of the pathogenesis of PE has been proposed internationally in recent years. In the first stage, which occurs during the first trimester, vascular remodeling disorders of uterine spiral arterioles caused by multiple factors result in “superficial placental implantation” and ultimately cause insufficient placental perfusion and impairment of placental function. In the second stage, which occurs during the second and third trimesters, the ischemic placenta will experience oxidative stress and release inflammatory factors, thus leading to systemic endothelial dysfunction. Therefore, patients with PE often have excessive inflammation of the immune system and an increased number of inflammatory factors in blood circulation. Jonsson Y et al. measured more than 20 different kinds of cytokines in serum and found significant differences in IL-6 and IL-8 between normal pregnant women and subjects with PE [16]. Sharma A et al. performed a similar study and found that compared with normal pregnant women, the levels of TNF-alpha, IL-6, and IL-8 in patients with PE were significantly increased [17]. The study of Catarino C et al. also indicated a significant increase in levels of IL-6, TNF-alpha, and C-reactive protein in patients with PE [18]. In contrast, several articles have shown that elevated proinflammatory cytokines, such as high-sensitivity C-reactive protein and IL-6, have been associated with the development of PPD [19, 20]. In addition, one study has noted that the development of depression and psychosis in young adulthood was associated with higher levels of IL-6 in childhood [21]. Therefore, increased inflammatory factors, such as C-reactive protein and IL-6, in the blood circulation of patients with PE may increase the incidence of PPD.

Finally, we attempted to explain the relationship between preeclampsia and PPD from the perspective of genetic changes since both of these diseases are caused by multiple factors and genes. A meta-analysis of 54 case-controlled studies with a total of 7,398 patients with PE and 11,230 normal pregnant women found that the 5,10-methylenetetrahydrofolate reductase (MTHFR) C677T genotype was a risk factor for PE [22]. Interestingly, PPD may also be accompanied by changes in the expression of certain genes, which has also been found in patients with PE. Li yin di et al. conducted a study on the relationship between PPD and genetic polymorphisms and found that MTHFR C677T and A1298C were independent risk factors for PPD [23], which may affect the incidence of PPD by changing the serum folic acid levels in women [24]. Some other genetic polymorphisms, such as 5-hydroxytryptamine transporter (5-HTT) and estrogen receptor (ESR), may also be related to increased susceptibility to PE and PPD [25–28]. Therefore, the abnormal expression of common genes such as MTHFR C677T, 5-HTT, and ESR may increase susceptibility to PPD in patients with PE.

We must admit that there are some limitations in our study. As a retrospective study, it suffered from bias. First, our study only included the target population in our hospital, lacking data from other regions. Patients with severe PE might be more likely to follow up in postpartum clinic, and this may result in selection bias. Second, in our study, the subjects included pregnant women with PE only. Those with other obstetric complications such as gestational diabetes mellitus (GDM) and intrahepatic cholestasis of pregnancy (ICP) were excluded. But as we all know, preeclampsia is closely related to some other complications such as GDM, as they share common high-risk factors such as obesity and advanced maternal age [29]. Equally, GDM is also a risk factor for PPD [30]: for pregnant women with PE and other complications associated with PE, if there was any change in the incidence of PPD; on the other hand, 93.3% (84/90) of patients with PE in our hospital choose to deliver via cesarean section to prevent blood pressure elevation as well as to prevent cardiovascular and cerebrovascular accidents secondary to the pain of childbirth; however women who deliver via cesarean section show an increased risk of PPD [4]; in other areas where cesarean section rates are not so high, if the prevalence of PPD will decrease; these are all causes of system error and require multicenter research. Third our diagnosis of PPD depends on EPDS scores, which has been widely used as a screening tool throughout the world rather than as a tool for clinical diagnosis by psychiatrists. And we performed questionnaire survey to collect information on the last 7 days; this could also lead to information bias. Fourth, we did not investigate annual household income, maternal education, family support, and other factors of women involved in the experiment, since they are all risk factors for PPD. Fifth, since our study is the first to discuss the relationship between PE and PPD, no other articles support our view. The several mechanisms we proposed are based on separate studies of PE and PPD. There were no targeted experiments to investigate whether there were differences in inflammatory factors and genetic changes in patients with PE and PPD. More research is required to explore the relationship between PE and PPD and its pathogenesis.

Despite some systematic errors, in our study, the prevalence of PPD in patients with PE was significantly higher than among normal women and, similarly, there was also an obvious difference in average score of EPDS between the two groups. So we identified PE as a risk factor for PPD. Youn, H. et al. performed a study on the relationship between obstetric risk factors and PPD and similarly found that PE increases the incidence of PPD [12]. But more researches are needed to support our view

## 5. Conclusion

In our study, the prevalence of PPD was 26.67% (24/90) in patients with PE, which was twice the prevalence of PPD among normal women (12.22%). Patients who had PE had nearly 3-fold increased odds for PPD compared to normal women. Patients with severe PE have a more than 4-fold higher risk of screening positive for PPD. Several mechanisms, such as clinical symptoms, inflammation, and genetic changes, have been used to explain the relationship between PE and PPD, but experiments are needed to prove these relationships. Since PE is an independent risk factor for PPD, additional prevention efforts and support methods should be provided for women with PE to reduce the incidence of PPD.

## Figures and Tables

**Table 1:** Comparison of demographic characteristics, delivery characteristics, and the proportion of preeclampsia in women with and without postpartum depression.

Variables	PPD group (N=35)	NO-PPD group (N=145)	*P* value
Normal women	11	79	
Preeclampsia	24	66	0.014
Delivery mode			
Vaginal delivery	4	32	
Cesarean section	31	113	0.158
Age(y)	30(27-33)	29(27-32)	0.621
Gravidity	2(1-4)	2(1-3)	0.905
Parity	0(0-1)	0(0-1)	0.516
Previous C-S*∗*	0(0-0)	0(0-0)	0.810
Gestational weeks(d)	268(262-275)	271(261.5-277)	0.289
Neonatal weight(g)	2900(2350-3410)	3020(2740-3300)	0.885
BMI (kg/m^2^)^#^	28.34(25.78-29.34)	28.23(26.04-30.32)	0.402
Premature birth			
Yes	7	33	
No	28	112	0.725

Continuous variables are presented as the median ± quartile, and the Mann-Whitney U test was used to determine differences. Categorical data are presented as frequencies, and the chi-square test was used to compare differences.

*∗*Previous cesarean section.

^#^BMI before delivery.

**Table 2:** Comparison of demographic characteristics and delivery characteristics in normal women.

Variables	PPD group (N=11)	NO-PPD group (N=79)	*P* value
Delivery mode			
Vaginal delivery	4	26	
Cesarean section	7	53	1.000
Age(y)	30(28-39)	30(27-32)	0.456
Gravidity	2(2-4)	2(1-3)	0.551
Parity	0(0-1)	0(0-1)	0.953
Previous C-S*∗*	0(0-0)	0(0-0)	0.530
Gestational weeks(d)	273(268-276)	275(271-278)	0.452
Neonatal weight(g)	3290(2800-3400)	3175(2950-3470)	0.530
BMI (kg/m^2^)^#^	28.35(24.80-29.34)	27.56(26.22-28.89)	0.460
Premature birth			
Yes	2	5	
No	9	74	0.203

Continuous variables are presented as the median ± quartile, and the Mann-Whitney U test was used to determine differences. Categorical data are presented as frequencies, and the chi-square test was used to compare differences.

*∗*Previous cesarean section.

^#^BMI before delivery.

**Table 3:** Comparison of demographic characteristics and delivery characteristics in patients with preeclampsia.

Variables	PPD group (N=24)	NO-PPD group (N=66)	*P* value
Severe preeclampsia	15	37	
Blood pressure			
<140/90 mmHg	22	63	
140/90~160/110 mmHg	2	3	
≥160/110 mmHg	0	0	0.862
Hypotensive			
Yes	6	18	
No	18	48	0.829
Clinical symptoms			
Yes	0	1(headache)	
No	24	65	1.000
Mode of delivery			
Vaginal delivery	0	6	
Cesarean section	24	60	0.293
Age(y)	29(27-32.75)	28.5(27-32.5)	0.912
Gravidity	2(1-3.75)	2(1-3.25)	0.771
Parity	0(0-1)	0(0-1)	0.290
Previous C-S*∗*	0(0-1)	0(0-1)	0.812
Gestational weeks(d)	265(262-274.25)	263(251.75-270.75)	0.361
Neonatal weight(g)	2760(2220-3410)	2825(2370-3210)	0.729
BMI (kg/m^2^)^#^	26.64(25.81-30.03)	29.28(25.98-31.44)	0.085
Premature birth			
Yes	5	28	
No	19	38	0.06

Continuous variables are presented as the median ± quartile, and the Mann-Whitney U test was used to determine differences. Categorical data are presented as frequencies, and the chi-square test was used to compare differences.

*∗*Previous cesarean section

^#^BMI before delivery.

**Table 4:** Comparison of EPDS scores between normal women and patients with preeclampsia.

Variables	Subjects (n)	EPDS scores	*t* test	*P *value
Normal women	90	4.42±3.320	Reference	Reference

Preeclampsia	90	7.27±3.136	-4.178	*P*<0.05

Severe preeclampsia	52	8.65±3.610	-5.011	*P*<0.05

**Table 5:** Multivariable logistic regression analysis for PPD.

	Adjusted OR	95% CI
Age (y)	0.954	0.855-1.064

Gravidity	0.728	0.520-1.020

Parity	1.320	0.432-4.037

Previous C-S*∗*	0.834	0.247-2.815

Gestational weeks (d)	1.018	0.970-1.068

Neonatal weight (g)	0.999	0.998-1.000

BMI (kg/m^2^)^#^	1.200	1.023-1.406

Preeclampsia	2.753	1.056-7.178

Severe preeclampsia	4.540	1.194-17.255

## Data Availability

The data used to support the findings of this study are included within the article.
